# EQ-5D self-reported health in Barbados and Jamaica with EQ-5D-5L population norms for the English-speaking Caribbean

**DOI:** 10.1186/s12955-021-01734-8

**Published:** 2021-03-19

**Authors:** Henry Bailey, Mathieu F. Janssen, Althea La Foucade, Girjanauth Boodraj, Marjorie Wharton, Philip Castillo

**Affiliations:** 1 Department of Economics, The University of the West Indies, St. Augustine Campus, St. Augustine, Trinidad and Tobago; 2 HEU, Centre for Health Economics, The University of The West Indies, St. Augustine Campus, St. Augustine, Trinidad and Tobago; 3 Arthur Lok Jack Global School of Business, The University of the West Indies, St. Augustine Campus, St. Augustine, Trinidad and Tobago; 4 Section Medical Psychology and Psychotherapy, Department of Psychiatry, Erasmus MC, Rotterdam, The Netherlands; 5 University of Technology, Kingston, Jamaica; 6 Sagicor Cave Hill School of Business and Management, The University of the West Indies, Cave Hill Campus, Cave Hill, Barbados; 7 University of Belize, Belmopan, Belize

**Keywords:** EQ-5D-5L, Barbados, Jamaica, Caribbean

## Abstract

**Background:**

The EQ-5D instrument is increasingly used in clinical and resource allocation decision making in developed and developing countries. EQ-5D valuation and population norms studies have been undertaken for Trinidad and Tobago, however no population norms or value sets have been generated for the other Caribbean countries. The aims of this study were to provide population norms for Barbados and Jamaica, and to develop a set of population norms that could be used by the other English-speaking Caribbean countries.

**Methods:**

The EQ-5D-5L self-reported health questionnaire was included in surveys of representative samples of adults in Barbados and adults in Jamaica in 2013. EQ-5D health states, mean EQ VAS scores and mean EQ-5D-5L index values (using the Trinidad and Tobago value set) were calculated for demographic groups in both countries based on 2347 respondents from Barbados and 1423 from Jamaica. A set of ‘Caribbean’ norms were developed by combining the Barbados and Jamaica data with norms recently published for Trinidad and Tobago.

**Results:**

Data were obtained for 2347 and 1423 respondents in Barbados and Jamaica respectively. The mean index and EQ VAS values were 0.943 and 81.9 for Barbados, and 0.948 and 87.8 for Jamaica. The health states most commonly observed in the two countries were similar. Generally the demographic patterns of self-reported health were consistent with those found in other studies. Some differences between the countries were observed in the patterns of rates of reporting problems on the EQ-5D dimensions among age-gender groups specifically for anxiety/depression and pain/discomfort

**Conclusion:**

This study has produced a set of EQ-5D population norms that can be used as base-line values in clinical and clinico-economic analyses for Barbados and Jamaica and for the English-Speaking Caribbean region.

**Supplementary Information:**

The online version contains supplementary material available at 10.1186/s12955-021-01734-8.

## Introduction

Economic evaluation continues to play an increasingly important role in resource allocation decision making [1]. This extends from clinical decision-making to decisions about the introduction and/or scaling up of interventions and services. Economic evaluation in healthcare necessitates estimating the relative costs and benefits associated with interventions. The Quality Adjusted Life Year (QALY) has become the most commonly used measure of the benefits associated with health interventions. The most commonly used multi attribute utility instrument for obtaining the quality adjustment for QALYs is the EQ-5D [2].

The EQ-5D instrument is used as a basis for the economic evaluation of health interventions in many developed countries [3]. A growing number of developing countries are also starting to apply EQ-5D. Countries such as Colombia [4], Poland and other Central European countries [5], Iran [6], and Malaysia [7], now recommend or require that EQ-5D-based preference weights be used in submissions for reimbursement or market access for medicines.

Clinicians and policy makers also use EQ-5D to track the progress of patients through an illness or treatment, and to compare the health status of different demographic groups. In order to use EQ-5D in ways such as these, clinicians, academics and policy makers often need reference or base-line values against which the health of different groups of people can be compared. These base-line values or’population norms’ reflect the health status of the general population [8].

Recently, EQ-5D population norms have been published for Trinidad and Tobago [9] however no EQ-5D population norms exist for any other Caribbean countries. The objectives of this study were to:

1. Provide EQ-5D population norms for Barbados and Jamaica.
2. Estimate a set of EQ-5D population norms for the English-Speaking Caribbean by combining the data for Barbados and Jamaica in this study with the data for Trinidad and Tobago published recently [9].

The EQ-5D system comprises five dimensions: mobility, self-care (the ability to bathe and dress oneself), usual activities (the ability to carry out usual activities such as work, study, leisure, etc.), pain/discomfort and anxiety/depression. In the 3-level EQ-5D instrument (EQ-5D-3L) each of these dimensions carries three levels of problems that a respondent can select in the EQ-5D questionnaire. These are ‘no problems’ (level 1), some or moderate problems (level 2), and extreme problems or unable to perform (level 3). Recently the EuroQol Group introduced a 5 level version of the instrument (EQ-5D-5L) to allow for greater sensitivity. The five dimensions are the same as in the 3 level instrument but two new levels have been added, resulting in the severity levels: no problems, slight problems, moderate problems, severe problems and extreme problems/unable to perform.

For several countries, EQ-5D index value sets are available. These are preference-based values that reflect the preferences of the population for each of the EQ-5D health states. These index values are used to make the quality adjustments in Cost Utility (or Cost per QALY) analysis. Recently, a value set was created for Trinidad and Tobago [10] but no value sets currently exist for any other Caribbean countries.

The EQ-5D instrument also includes a self-assessed rating in which a respondent provides their own assessment of their health on a 0–100 visual analogue scale (with the anchors worst imaginable health at 0 and best imaginable health at 100). These are referred to as EQ VAS values and (along with the preference based index values) they are used to compare different groups of patients or to track the progress of patients through the course of an illness or a treatment.

## Methods

The EQ-5D-5L self-reported health questionnaire was included in the 2013 cycle of the Adult Population Survey (APS) of the Global Entrepreneurship Monitor (GEM) Study for Barbados and Jamaica. For countries that participate in the GEM Study, the APS is a survey of a representative sample of at least 2000 citizens. This survey includes attitudes, perceptions, and aspirations among the general population about entrepreneurship. Details of the GEM study are provided elsewhere [11].

In Barbados 2355 respondents (aged 18–64) completed the APS. EQ-5D-5L data were collected from 2353 of these. Respondents were stratified based on age, gender, and parish using quota sampling. The GEM Report for Barbados reports data for 2302 respondents [12]. This is because 53 respondents were dropped for the GEM study due to missing data concerning important GEM-related questions. These respondents were included in this population norms study since their demographic and EQ-5D-5L health data were collected. For the face to face interviews in Barbados, one respondent was randomly selected from each household by the interviewers.

In Jamaica, a multi-stage, stratified probability sampling procedure was used to select 2246 respondents, aged 18–64 years, who lived in private dwellings and resided permanently in Jamaica. The first stage of stratification was at the level of the parish while the second stage involved the selection of rural and urban areas in each parish. The numbers of rural and urban sampling units selected in each parish were proportional to the total number of rural and urban districts in the respective parishes. Finally, the primary sampling units (PSUs) were chosen from the selected rural and urban districts. The PSUs are enumeration districts (EDs) with 80 or more dwellings, according to the 2011 Population Census. The EDs and maps were obtained from the Statistical Institute of Jamaica. All of the interviews were conducted face-to-face, during the day, evenings and on weekends, with a maximum of five callbacks to each dwelling. Respondents were selected using the ‘next birthday rule’ and interviewers were trained in terms of the purpose of the survey, interviewing strategies and protocols, and relevant ethical procedures guiding data collection. Of the 2246 respondents in Jamaica, EQ-5D-5L data were collected from 1498, so the sampling weights were not used in this study.

The EQ-5D-5L questionnaire was included in the 2012/2013 cycle of the GEM APS for Trinidad and Tobago. While population norms have been presented for Trinidad and Tobago elsewhere [9] the data from the Trinidad and Tobago study were combined with data from Barbados and Jamaica in this study to produce a set of population norms for the English-Speaking Caribbean based on data from these three countries. The Trinidad and Tobago survey included 2036 respondents aged 18 and over from all administrative areas of the country based on population and age-gender breakdowns based on the most recent census data. Enumeration districts were selected from enumeration district maps of the Central Statistical office of Trinidad and Tobago and one in every four households were visited in each selected enumeration district. Respondents were selected using the most previous birthday rule. Call back cards were left at the household when the selected respondent was not at home. In all three countries, the samples excluded individuals who lived in institution-type residences such as army camps, prisons, school dormitories, and guest houses. Respondents were not paid in these surveys.

Some inconsistencies were observed in the data. There were 30 observations of state 55555 (extreme problems on all 5 dimensions) with EQ VAS values of 100. This was considered to be inconsistent. 27 of these observations were from one interviewer in Jamaica. This interviewer had another 5 respondents with state 55555 and EQ VAS values of 95 and over. This was most likely to be the result of data entry error or misunderstanding on the part of respondents. All 69 observations from this interviewer were removed. There were 9 other observations of 55555 in Jamaica from other interviewers. Five of these had EQ VAS values between 80 and 99, and the other two had no VAS values. All of these respondents listed their work status as ‘working’. These nine observations were also excluded. A decision was taken to extend exclusion criteria to observations with EQ VAS values of over 90 and two or more EQ-5D dimensions at level 4 or 5 on the basis of inconsistency. There were 22 such observations. (1 in Barbados, and 21 in Jamaica). Half of these reported living alone. The Satisfaction With Life Scale [13] was also included in the GEM APS. More than half of these respondents reporting state 55555 strongly agreed with the statements*: “In most ways, my life is close to my ideal” and “The conditions of my life are excellent”*. The final dataset thus comprised 2347 in Barbados and 1423 in Jamaica. These exclusions mean that the samples would no longer be strictly representative of the countries, although the total numbers of exclusions are relatively small. The GEM Reports for all three countries are available online [14].

For each respondent in the three countries, data were collected on the level of each EQ-5D dimension (thereby providing the EQ-5D-5L state for the respondent), the respondent’s EQ VAS value, and demographic data. The demographic variables included in the APS were: age, gender, district or parish of residence, employment status, education level, whether they had private health insurance, income group, ethnicity, and household size. Employment status was trichotomized in to three groups: working, unemployed, and those who choose not to work. Income was trichotomized into upper, middle and lower thirds. Ethnicity was not included in the Jamaica survey. The private health insurance question was not included in the Barbados survey.

The district or parish of residence was dichotomized differently for each country. For each country, consideration was given to finding a geographic dichotomy that would be most likely to reveal a difference in health status. Thus for Barbados, the respondents were split into two groups: the parish of St. Michael and all other parishes. St Michael includes the capital city, the university, and the two hospitals on the island and is considered to be more developed than other parishes. Jamaica is much larger than Barbados, and respondents were grouped into urban and rural categories. Health status was expected to be higher in urban centres in Jamaica because the major health and other facilities are located in urban centres- For each country, respondents were grouped geographically such that Group I included areas where health status was expected to be higher, and Group 2 included areas where health status was expected to be lower.

The EQ-5D-5L instrument allows for 5^5^ = 3125 possible states. The analysis began with a profile of the states making up 90% of the sample in each country and the percentages of respondents in each of those states.

The mean VAS values, EQ-5D index values and ceiling levels were calculated for each sub group of each of the demographic variables. The ceiling level is the percentage of respondents who report no problems on all 5 dimensions of the EQ-5D instrument (state 11111).

Differences in EQ VAS and index values among demographic groups within each country were tested for statistical significance using t-tests (where for a given variable there were only two alternatives, such as gender) and ANOVA. Such differences were considered to be statistically significant if the associated p-values were 0.05 or less. Given the importance of age and gender as drivers of self-reported health [8], the analyses were repeated for age and gender groups for each country.

The analyses were also carried out using aggregated data for Barbados, Jamaica, and data recently published for Trinidad and Tobago. This combination can be used as a set of population norms for the English-Speaking Caribbean region. These can be used as baseline values for other Caribbean countries until population norms studies can be conducted in those countries. To create the Caribbean norms, country index, EQ VAS, ceiling percentages and problem reporting rates were weighted by the relative populations of each country at gender-age sub group level using data obtained from the statistical office of each country for 2013. For example, in the 18–24 age group, the number of males totalled 12,398 in Barbados, 186,857 in Jamaica and 78,273 in Trinidad and Tobago. To obtain the weighted mean, the mean EQ VAS value for this demographic group from each island was multiplied by the respective number of males aged 18–24 divided by the sum of all three of these totals (277,528). This process was used for all age and gender groups and age-gender sub groups. For variables other than age and gender, weights were calculated based on the total population of each country in 2013.

The relationships between the socio-economic variables included in the GEM survey and the EQ VAS and index values were further investigated using a regression model to control for age and gender which are known to be major drivers of EQ VAS and index values. Because of the skewed distributions of the dependent variables, a Generalized Linear Model (GLM) with a Poisson distribution and a log link was used [15, 16]. Because some index values were negative, a disutility score (1 – index value) was used as the dependent variable for the index value GLMs.

There is no EQ-5D value set for Barbados or Jamaica. The Trinidad and Tobago value set [10] was used to provide index values for the EQ-5D states in Barbados and Jamaica in this study. Caution should be exercised in using EQ-5D index values from one country in another [17]. The Trinidad and Tobago EQ-5D value set was developed using the EQ-5D-3L version, however the instrument that was included in the GEM survey for Barbados and Jamaica was the 5 level version. To facilitate the use of the Trinidad and Tobago index values for this dataset, the EQ-5D-3L index values were transformed via a crosswalk algorithm to map them onto 5 level values [18].

All analyses were conducted using STATA 14 (Stata Corporation, College Station, Texas).

## Results

The demographic composition of the respondents from Barbados, and Jamaica, along with the Caribbean combination are presented in Table 1. Some of the sub groups in Table 1 will not add up to the total numbers of respondents in the respective countries. For example, gender was not captured for 6 respondents in Barbados. Some respondents were not willing to divulge their incomes. Some respondents were not aware of whether they were covered under private health insurance.

**Table 1:** Socio-demographic characteristics of the sample

	Barbados	Jamaica	Caribbean^a^
*Total respondents*	2347	1423	5806
*Age group*			
18–24	409	216	946
25–34	556	413	1435
35–44	560	364	1287
45–54	492	282	1145
55–64	288	148	702
> 64	N/A	N/A	248
*Gender*			
Male	997	726	2724
Female	1344	697	3076
*Health insurance*			
Yes	N/A	415	873
No	N/A	1008	2565
*Employment*			
Working	1734	1308	4315
Choose not to work	51	32	335
Unemployed	236	49	395
*Region in country*			
I	797	727	N/A
II	1546	696	N/A
*Ethnicity*			
Afro	2209	N/A	N/A
Indo	51	N/A	N/A
Other	72	N/A	N/A
*Income*			
Lower 33.3%	465	225	1400
Middle 33.3%	378	247	1113
Upper 33.3%	456	175	1044
*Education*			
Incomplete Secondary or less	244	244	1104
Complete Secondary or Vocational	1903	1011	3947
Tertiary	186	159	722

^a^Caribbean = Barbados, Jamaica and Trinidad & Tobago

216 EQ-5D-5L states were observed in this study. For each country, and for the ‘Caribbean group’, 12 states accounted for 90% or more of the sample. Similarities between the health profiles of the countries in this study are shown in Table 2.

**Table 2:** The 12 most commonly observed states in each country and in the Caribbean combination

Barbados			Jamaica			Caribbean^a^		
State	%	Cumulative %	State	%	Cumulative %	State	%	Cumulative %
11111	66.4	66.4	11111	68.9	68.9	11111	68.8	68.8
11121	9.2	75.6	11121	6.2	75.1	11121	7.7	76.5
11112	4.2	79.8	11112	5.4	80.5	11112	4.2	80.7
11122	2.8	82.6	11122	4.2	84.7	11122	2.6	83.3
11131	2.5	85.1	11123	0.9	85.6	11131	1.5	84.8
21121	1.3	86.4	11211	0.8	86.4	21121	1.4	86.2
11113	1.0	87.4	11114	0.8	87.2	11113	0.8	87.0
11211	0.8	88.2	11221	0.8	88.0	11211	0.7	87.7
21111	0.6	88.8	21111	0.8	88.8	21111	0.6	88.3
11141	0.5	89.3	11113	0.6	89.4	21221	0.5	88.8
11221	0.5	89.8	21121	0.6	90.0	11221	0.5	89.3
11123	0.5	90.3	21122	0.5	90.5	11123	0.5	90.2

^a^Caribbean = Barbados, Jamaica and Trinidad & Tobago

**Fig. 1:**
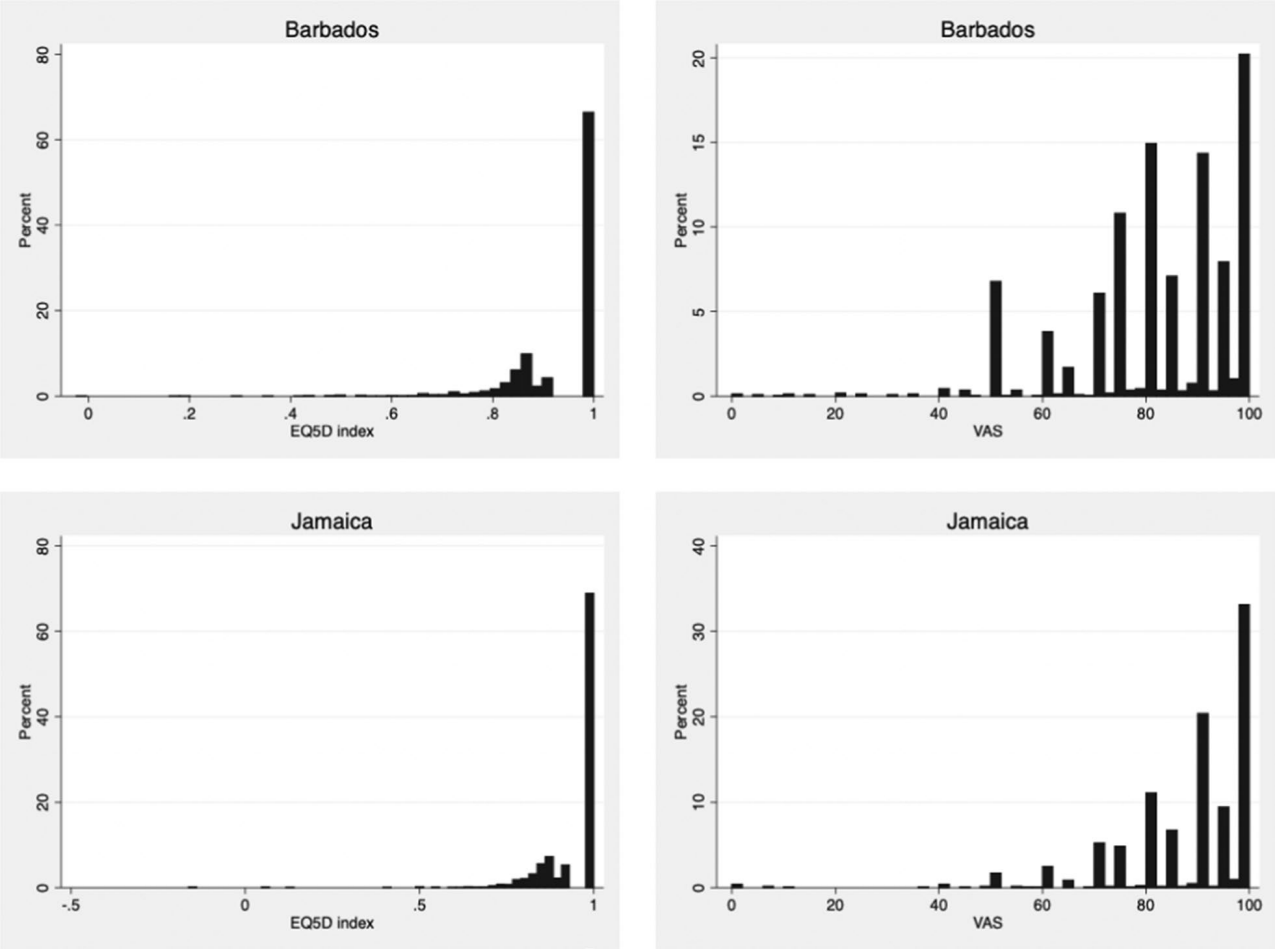
The distributions of EQ-5D-5L Index values and EQ VAS values for each country

The distributions of Index and EQ VAS scores for each country are presented in Fig. 1. The mean index and EQ VAS values were 0.943 and 81.9 for Barbados, and 0.948 and 87.8 for Jamaica. Index values ranged from − 0.024 to 1.000 (Barbados) and − 0.163 to 1.000 (Jamaica) while EQ VAS values ranged from 0 to 100 in both countries.

Additional file 1 shows the mean EQ VAS and index values by age group and gender for each country and for the combined Caribbean set.

Additional file 2 shows the mean EQ VAS values, EQ-5D-5L index values and ceiling effects by demographic group for each country and the combined values (weighted by population and unweighted) for the Caribbean group: Trinidad and Tobago, Barbados and Jamaica.

The variables in Additional file 2 failed Bartlett’s test of equality of variances among the subgroups, so Welch’s ANOVA and Welch’s t-test were used, as these tests allow for unequal variance. The p-values in Additional file 2 show the significance of the Welch’s ANOVA and t-test statistics. To allow for multiple comparisons, a Bonferroni correction was applied to the p values associated with the ANOVA tests. Thus, both of the countries and the Caribbean region in Additional file 2 have p-values of 0.000 for index values by age group so the differences in these variables among the age groups are highly significant. The declining ceilings as age rises reflect lower health status in higher age groups. For the weighted VAS and EQ-5D Index values, standard errors were calculated using the formula:

$$S.E. = \left( {\sqrt {\frac{{{\Sigma }_{i = 1}^{c} \left( {n_{i} - 1} \right)s_{i}^{2} + {\Sigma }_{i = 1}^{c} n_{i} \left( {\overline{x}_{i} - \overline{x}} \right)^{2} }}{{N_{total} - 1}}} } \right) \div \surd {\text{n}}_{{\text{i}}}$$

where n is the sample size, s^2^ is the variance and c is the number of countries. The p-values associated with the ANOVA tests on the weighted VAS and EQ-5D index values were taken from the ANOVA tests for the unweighted VAS and EQ-5D Index values.

Differences in EQ VAS and index values between males and females were significant to the 5% level for both countries with males having higher values on both measures. The ceiling was also higher for men than for women in both countries. Having private health insurance was associated with a higher ceiling, index and EQ VAS values and the difference was significant to the 5% level.

Having a job was found to be associated with a higher ceiling, index and EQ VAS values than being unemployed in Barbados but not in Jamaica.

In both countries the mean EQ VAS value was actually higher in Region II, but this difference was only significant to the 5% level in Barbados. The ceiling was higher in Region I than in Region II for Jamaica, but lower in Region I for Barbados. Ceiling, index and EQ VAS values increased with income in Barbados (significant to the 5% level for index values) while in Jamaica, the middle income group had the highest levels on all three measures but the differences for index and EQ VAS values were not significant to the 5% level.

Education was found to be associated with differences in index and EQ VAS values that were significant to the 5% level except for EQ VAS values in Barbados. The lowest education group (incomplete high school) consistently had the lowest mean EQ VAS, index values and ceiling levels.

Household size was found to be significantly (to the 5% level) associated with index values in Jamaica with people living alone having the highest mean index value and ceiling level.

Additional file 2 also provides weighted ceiling, index and EQ VAS values for a combination of Barbados, Jamaica and the recently published Trinidad and Tobago values. For the weighted averages, age, gender, health insurance, employment status and education level all have p values of less than 0.05 for both EQ VAS and index values. In the case of employment status, the substantially higher health status of the unemployed group in Jamaica carried over into the weighted average. Differences were significant to the 5% level for index values in income groups and EQ VAS values in ethnic groups.

To observe the effect of weighting by population, Additional file 2 also includes unweighted averages for ceiling, index and EQ VAS values. The differences between the weighted and unweighted ceiling levels were all of the order of 2% except for employment (choose not to work had a weighted average ceiling that is 5.4% lower than the unweighted average and unemployed had a 9% higher weighted average ceiling than the unweighted average). Also, for afro- and household size of 1, the weighted ceilings were approximately 4.5% higher. All of the weighted index values were within 2% of the unweighted index values except for household size of 2–3 where the weighted average index value was 7% lower than the unweighted value. The weighted EQ VAS values were generally of the order of 3% (or less) higher than the unweighted averages except for the ‘Choose not to work’ group where the weighted mean EQ VAS was actually 1% lower and unemployed where the weighted mean EQ VAS was 7% higher than the unweighted mean.

**Fig. 2:**
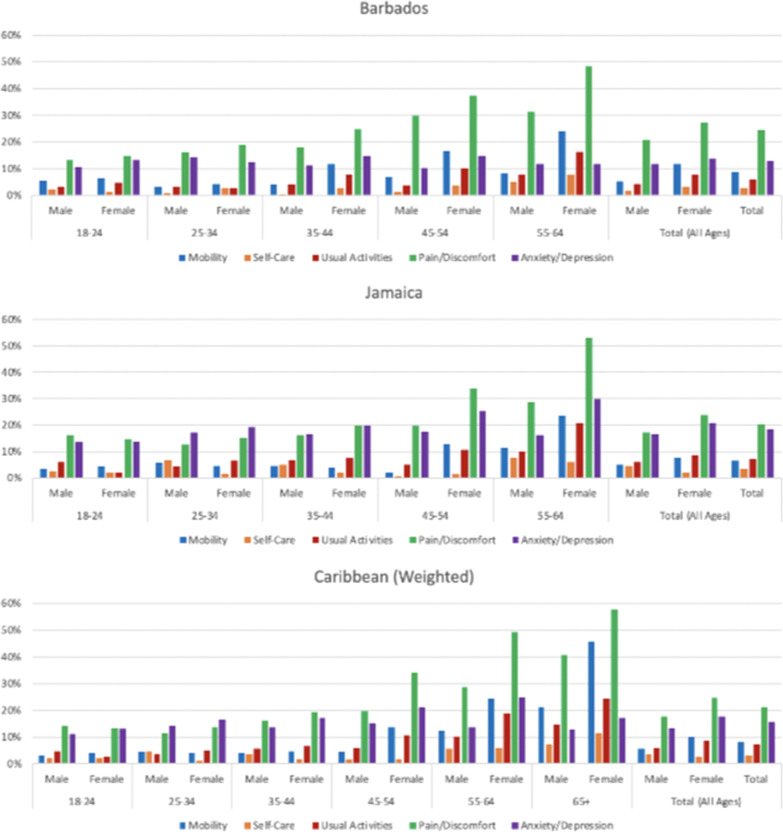
Rates of reporting problems at any level (2 through 5) for the EQ-5D dimensions by age and gender

The frequencies of reporting problems at each level on each dimension are summarized graphically in Fig. 2 and detailed response rates are presented in Additional file 3.

For the graphs in Fig. 2, the responses are dichotomized so the values on the vertical axes show the percentages of respondents in each age-gender group who reported problems at any level (2 through 5) on each dimension. The patterns of problem reporting across age-gender groups for the two countries were generally similar with pain/discomfort and anxiety/depression being the dimensions to have the highest frequency of reported problems, and with frequency increasing with age. Males in Barbados reported problems with pain/discomfort more frequently than males in Jamaica in all age groups beyond 18–24. Problems with anxiety/depression were more prevalent across all age-gender groups in Jamaica than in Barbados.

In Barbados and Jamaica, higher percentages of women reported problems with pain and mobility than men. This disparity increased with age. In Jamaica the rate of reporting anxiety/depression also increased for women as they age. This was not observed in Barbados.

The effects of demographic variables on EQ VAS and index values (using disutility as the dependent variable) in Additional file 2 were tested for statistical significance at country level with controls for age and gender. The GLM results for EQ VAS are presented in Additional file 4. In Barbados, ‘having lower income’ being unemployed and ‘lower educational attainment’ were found to have statistically significant effects on EQ VAS values. Having lower educational attainment was found to have a positive effect on EQ VAS values in this model. In Jamaica, unemployment was found to have a positive and significant effect on EQ VAS value. None of the demographic variables were observed to have a significant effect on the index values in the GLM analyses.

## Discussion

This is the first study to report EQ-5D-5L data for Barbados and Jamaica, and to produce a set of EQ-5D-5L population norms for the English-Speaking Caribbean region. Generally, the findings along with those reported for Trinidad and Tobago [9] suggest that people in the English-Speaking Caribbean region report relatively high health related quality of life as captured by the EQ-5D descriptive system when compared to other countries.

The country-level index mean values of 0.943 and 0.948 in Additional file 2 are higher than those of 0.89–0.92 reported in other countries [14, 19–21]. This could be due to the index values in this study all being based on the same value set from Trinidad and Tobago. It could also be due to the high ceiling levels in in the three Caribbean countries (all over 66%) compared to ceiling levels (below 50%) observed in other EQ-5D-5L population norms studies [14, 19–22] with only three other countries showing ceiling levels of 54–62% [23–25].

The mean EQ VAS Values of 81.9 and 87.8 observed in this study were also high relative to the EQ VAS values of 71.1 to 83.7 reported in one review of 18 countries [8]. This could be related to the high percentages of respondents in this region who report EQ VAS values of 100. Analysis of data from population norms studies in 18 countries included in that review show that this ratio is in the 5–15% range with only one country (Sweden) as an outlier just below 20% [8]. In our study, for Barbados 14% of the respondents reported EQ VAS values of 100. However, in Jamaica and Trinidad & Tobago, 30% and 22% of the respondents respectively reported EQ VAS values of 100. Further investigation may be warranted in to the high proportion of respondents reporting EQ VAS values of 100 in Trinidad & Tobago and Jamaica, as well as the high ceiling levels in all 3 Caribbean countries.

The distributions of index values in Fig. 1 follow the patterns observed in other countries [8] being negatively skewed with the majority of the respondents having a value of 1. The EQ VAS values also generally followed the pattern observed in many other countries- also being negatively skewed with values clustered around multiples of 10 and 5 (80, 85, 90, 95 etc.). Among the respondents who gave their health state as 11111 (no problems on any dimension and therefore index value of 1.000) the percentages giving EQ VAS scores of 100 (best health imaginable) were 17% in Barbados, and 37% in Jamaica For the ‘Caribbean’ set (which includes Trinidad and Tobago) this figure is 26%. Extending this analysis, the percentage of respondents with state 11111, reporting EQ VAS scores of 95 or higher was 33% and 51% for Barbados and Jamaica respectively, and 40% for the Caribbean combination. Different patterns are usually observed for EQ VAS and EQ-5D index values as these two measures capture different things [26]. The index value captures the societal hypothetical value for a given EQ-5D-5L state, whereas the EQ VAS value captures the overall self-assessment of an individual’s health at the time of the elicitation exercise. The latter will include factors that are external to the EQ-5D dimensions. While both measures are useful for measuring or tracking the health status of patients, patient groups or demographic groups, the index values are elicited using ‘choice based’ valuation methods which make them suitable for resource allocation decision making (e.g. for Cost per QALY analysis, 10).

The similarity in EQ-5D-5L health profiles among the countries that were included in this study is evident in the states observed in Table 2. Both countries, and the Caribbean set had the top four states in the same order. Other than 11131 and 11141 in Barbados, 11114 and 21122 in Jamaica the two islands had the same states in their 12 most commonly reported states.

The rates of reporting problems on the EQ-5D dimensions also showed some differences in patterns between the countries in this study as seen in Fig. 2. Further research could be undertaken to investigate the drivers of pain and mobility problems among women that were observed in Barbados and Jamaica and the higher levels of anxiety/depression that were observed in Jamaica. The issue of the higher prevalence of pain and mobility problems among women than men in higher age groups has been investigated elsewhere. These differences have been attributed to differences in perception [27], higher prevalence of musculoskeletal, mental and other pain disorders [28], fibromyalgia [29] among women, and other biologic and psychosocial factors [30]. The differences in the patterns of problems reported on the EQ-5D dimensions across the three countries in this study may warrant further investigation.

There are some limitations to this study. One limitation is the exclusion of marital status as this is known to affect health status [8]. This study applied the EQ-5D index values from Trinidad and Tobago to the other two Caribbean islands. While the Trinidad and Tobago index values may not exactly reflect the health state preferences of Barbados and Jamaica, this approach of using index values from a nearby/culturally similar country has been recommended where no local EQ-5D value set exists [3]. Further the Trinidad and Tobago EQ-5D-5L value set is the result of a crosswalk algorithm applied to the EQ-5D-3L value set. This follows the recommendation for countries that have an EQ-5D-3L value set but no EQ-5D-5L value set, and for which researchers wish to attach index values to EQ-5D-5L states [18]. Crosswalk values sets have been used in several studies in such countries [31–36] and in at least one case a crosswalk value set is recommended even  though an EQ-5D-5L value set is available [37]. The EQ-5D-5L crosswalk value set for Trinidad and Tobago embodies the same preference patterns among its domains as the EQ-5D-3L value set.

Finally, a cut off at age 64 that was applied in the Barbados and Jamaica GEM surveys. This prevented the study from capturing data for higher age groups.

## Conclusion

This study has reported the first set of EQ-5D population norms for Barbados and Jamaica, and it has also provided a set of EQ-5D population norms that can be used in other English-Speaking Caribbean countries. The data provided can be used as base line values by researchers in the English-Speaking Caribbean until population norms data can be obtained for the rest of the region. The results are generally consistent with findings of population norms studies from other countries: with most respondents self-reporting as being in full health, men having higher self-reported health than women, and with self-reported health declining as respondents age.

## Supplementary Information

**Additional file 1:** Microsoft Excel .xlsx file showing the mean EQ VAS and index values by age group and gender for each country and for the Caribbean combination.

**Additional file 2:** Microsoft Excel .xlsx file showing the mean EQ VAS values, EQ-5D-5L index values and ceiling effects by demographic group for each country and the Caribbean combination.

**Additional file 3:** Microsoft Excel .xlsx file showing the frequencies of reporting problems at each level on each dimension for each country and for the Caribbean combination.

**Additional file 4:** Microsoft Excel .xlsx file showing generalized linear model of the effects of demographic variables on EQ VAS values with controls for age and gender.

## Data Availability

The data that support the findings of this study are the Global Entrepreneurship Monitor datasets for these countries. These datasets are available from the respective authors upon reasonable request from each country: Barbados (MW), and Jamaica (GB). Requests can be routed through the corresponding author (HB).
